# Structures of the *Plasmodium falciparum* heat-shock protein 70-x ATPase domain in complex with chemical fragments identify conserved and unique binding sites

**DOI:** 10.1107/S2053230X21007378

**Published:** 2021-07-28

**Authors:** Nada Mohamad, Ailsa O’Donoghue, Anastassia L. Kantsadi, Ioannis Vakonakis

**Affiliations:** a Department of Biochemistry, University of Oxford, South Parks Road, Oxford OX1 3QU, United Kingdom

**Keywords:** *Plasmodium falciparum*, PfHsp70-x, heat-shock proteins, malaria, chaperones, erythrocyte remodelling, crystallography, complexes, fragment screening

## Abstract

*Plasmodium falciparum* invades erythrocytes and extensively modifies them in a manner that increases the virulence of this malaria parasite. A single heat-shock 70 kDa-type chaperone, PfHsp70-x, is among the parasite proteins exported to the host cell. PfHsp70-x assists in the formation of a key protein complex that underpins parasite virulence and supports parasite growth during febrile episodes. Previous work resolved the crystallographic structures of the PfHsp70-x ATPase and substrate-binding domains, and showed them to be highly similar to those of their human counterparts. Here, 233 chemical fragments were screened for binding to the PfHsp70-x ATPase domain, resulting in three crystallographic structures of this domain in complex with ligands. Two binding sites were identified, with most ligands binding proximal to the ATPase nucleotide-binding pocket. Although amino acids participating in direct ligand interactions are conserved between the parasite and human erythrocytic chaperones, one nonconserved residue is also present near the ligand. This work suggests that PfHsp70-x features binding sites that may be exploitable by small-molecule ligands towards the specific inhibition of the parasite chaperone.

## Abstract 2

233 chemical fragments were screened for binding to the ATPase domain of the Hsp70-x chaperone from the malaria parasite *Plasmodium falciparum*. Crystallographic structures of this domain in complex with different fragments revealed a major binding site proximal to the ATPase catalytic pocket. It is shown that a residue near this binding site is not conserved between *P. falciparum* and human erythrocytic Hsp70 chaperones, which may provide a basis for specific inhibition of the parasite enzyme.

## 1. Introduction   

Over 400 000 deaths are attributed to malaria each year, primarily in sub-Saharan Africa and among children under the age of five years (World Health Organization, 2019 ▸). Nearly 95% of these deaths are caused by a single parasite species, *Plasmodium falciparum*. Compared with other human-infective plasmodia, *P. falciparum* induces a strong virulence phenotype in erythrocytes it invades, which is responsible for severe disease. The parasite exports a complement of nearly 500 proteins to the host cell (Spillman *et al.*, 2015 ▸; Warncke *et al.*, 2016 ▸; Proellocks *et al.*, 2016 ▸; de Koning-Ward *et al.*, 2016 ▸), among which is the adhesion ligand *P. falciparum* erythrocyte membrane protein 1 (PfEMP1; Hviid & Jensen, 2015 ▸). PfEMP1 localizes to the parasitized erythrocyte surface, where it integrates into large ‘knob’-like complexes (Cutts *et al.*, 2017 ▸). The formation of knob-like structures is unique to *P. falciparum*-infected erythrocytes and leads to parasitized cells adhering to the microvasculature of organs such as the brain and kidneys (‘cytoadhesion’), as well as the formation of cell clumps between erythrocytes (‘rosetting’). Cytoadhesion and rosetting are beneficial to the parasite as they allow the avoidance of splenic passage, where parasitized erythrocytes may be recognized and destroyed, thereby enhancing parasite growth. However, these properties are deleterious to the host, as they cause blockage of blood vessels, tissue damage from inflammation and oxygen deprivation, and can lead to coma and death (Craig *et al.*, 2012 ▸; Smith *et al.*, 2013 ▸).

A single heat-shock 70 kDa-type chaperone, PfHsp70-x (PF3D7_0831700), is present among the parasite proteins exported to the host (Külzer *et al.*, 2012 ▸). Although PfHsp70-x has been shown to be dispensable for parasite viability in cell cultures, removal of this protein reduced the efficiency of PfEMP1 transport to the erythrocyte surface and consequently decreased the cytoadherence of parasitized cells under simulated blood-flow conditions by ∼60% (Charnaud *et al.*, 2017 ▸). Further, PfHsp70-x, in concert with Hsp40-type co-chaperones exported by the parasite (Külzer *et al.*, 2012 ▸; Day *et al.*, 2019 ▸; Dutta *et al.*, 2021 ▸), is thought to contribute more generally to maintaining homeostasis of proteins exported to the host cell, akin to the general role of Hsp70-type chaperones across the kingdoms of life (Meimaridou *et al.*, 2008 ▸). The parasite export machinery, comprising a large translocon complex (PTEX; Ho *et al.*, 2018 ▸), may also be assisted by PfHsp70-x as the chaperone has been shown to associate with PTEX in cells (Zhang *et al.*, 2017 ▸). Consistent with a broader role for PfHsp70-x in *P. falciparum* biology, depletion of this chaperone reduced parasite growth by ∼40% during heat-shock conditions that simulated febrile episodes of malaria patients (Day *et al.*, 2019 ▸).

Hsp70 chaperones comprise an N-terminal ATPase domain connected by a short linker to a C-terminal substrate-binding domain (Boorstein *et al.*, 1994 ▸; Meimaridou *et al.*, 2008 ▸). ATP hydrolysis triggers conformational changes in the chaperone that cause sequential binding, holding and release of exposed hydrophobic segments in target proteins. Crystallographic structures of both the ATPase and substrate-binding domains of PfHsp70-x have been resolved, and showed a very high degree of structural similarity to the equivalent domains of human erythrocytic chaperones (Schmidt & Vakonakis, 2020 ▸; Day *et al.*, 2019 ▸). Despite this similarity, bioinformatics analysis suggested that the PfHsp70-x ATPase domain may possess ‘druggable’ sites for small-molecule interactions that are structurally distinct compared with the equivalent sites in human counterparts (Day *et al.*, 2019 ▸). To test this prediction, we screened chemical fragments for binding to the PfHsp70-x ATPase domain using crystallographic means (Erlanson *et al.*, 2016 ▸). Here, we report the identification of two small-molecule interaction sites on the ATPase domain of PfHsp70-x and compare these sites across human and parasite Hsp70 chaperones.

## 2. Materials and methods   

### 2.1. Macromolecule production   

Production and purification of the PfHsp70-x ATPase domain was performed as described previously (Day *et al.*, 2019 ▸). Briefly, a genetic construct encoding PfHsp70-x residues 29–419 in a pFloat vector (N-terminal His_6_ tag, HRV 3C protease cleavage site; Rogala *et al.*, 2015 ▸) was transformed into *Escherichia coli* BL21(DE3) cells (Table 1 ▸). The cells were grown in Luria–Bertani medium at 310 K; protein expression was induced using 0.25 m*M* isopropyl β-d-1-thiogalactopyranoside and was allowed to proceed at 291 K for 18 h. The cells were resuspended in lysis buffer [10 m*M* Na_2_HPO_4_, 2 m*M* KH_2_PO_4_, 2.7 m*M* KCl, 500 m*M* NaCl, 0.01%(*v*/*v*) Triton X-100, 5 m*M* ATP, 2 m*M* MgCl_2_ pH 7.4] and disrupted by sonication, and the PfHsp70-x ATPase domain was initially purified using a HiTrap TALON metal-affinity column (GE Healthcare Life Sciences). Following cleavage of the His_6_ tag using HRV 3C protease, further purification was performed by size-exclusion chromatography on a Superdex 75 column (GE Healthcare Life Sciences) equilibrated in crystallographic buffer [20 m*M* 4-(2-hydroxyethyl)-1-piperazine­ethanesulfonic acid (HEPES) pH 7.4, 50 m*M* NaCl, 1 m*M* DTT]. The protein was concentrated by spin ultrafiltration and the concentration was calculated by UV absorption at 280 nm using an extinction coefficient estimated from the amino-acid sequence (Pace *et al.*, 1995 ▸).

### 2.2. Crystallization and fragment-screening setup   

Diffraction-quality crystals were obtained in large numbers using the sitting-drop vapour-diffusion method (Table 2 ▸) in trays set up using a Mosquito robot (TTP Labtech). Crystals appeared in under two days and continued to evolve for a total of 7–10 days.

Crystal soaking with solutions of fragments was performed using the integrated pipeline in the XChem laboratory at Diamond Light Source. An initial evaluation showed that the majority of PfHsp70-x ATPase domain crystals retained good diffraction characteristics following the addition of up to 20%(*v*/*v*) dimethyl sulfoxide (DMSO) to the crystallization drop and incubation for up to 3 h. Fragments from the DSI-poised (Cox *et al.*, 2016 ▸) and FragLite (Wood *et al.*, 2019 ▸) chemical libraries at 500 m*M* concentration in DMSO were added to the crystallization drops to a final concentration of 50.6 m*M* [10%(*v*/*v*) final DMSO concentration] using an Echo liquid-handling robot (Beckman Coulter). The crystals were incubated with fragments for 2–3 h prior to harvesting and rapid cooling in liquid nitrogen. A list of the fragments tested is given in Supplementary Table S1.

### 2.3. Data collection and processing   

Diffraction data were collected on Diamond Light Source (DLS) beamline I04-1 at 100 K to typical resolutions of 2.0–2.6 Å. The data were processed using *xia*2 (Winter *et al.*, 2013 ▸). The space group was determined to be *P*2_1_2_1_2_1_, with two copies of the PfHsp70-x ATPase domain per asymmetric unit. The data sets were analysed using *PanDDA* (Pearce *et al.*, 2017 ▸) for the presence of bound ligands (Supplementary Table S1), using a single copy of the crystallographic structure of the PfHsp70-x ATPase domain (PDB entry 6rzq, chain *A*; Day *et al.*, 2019 ▸) as a reference and a molecular-replacement model. Molecular replacement and initial evaluation of unmodeled electron density was performed by *DIMPLE* (Wojdyr *et al.*, 2013 ▸). Data-set statistics for crystals that were evaluated to have bound ligands are shown in Table 3 ▸.

### 2.4. Structure solution and refinement   

Structure refinement was performed using *BUSTER* (Bricogne *et al.*, 2017 ▸) starting from the complete crystallo­graphic model of the PfHsp70-x ATPase domain (PDB entry 6rzq; Day *et al.*, 2019 ▸), as the data sets were isomorphous in all cases to this earlier model. Model fitting was performed in *Coot* (Emsley *et al.*, 2010 ▸). Fractional ligand occupancies were refined automatically in *BUSTER*. In the structure with PDB code 7p31, the fractional occupancy of the ligand I atom was refined separately from the rest of the fragment. Model validation was performed using *MolProbity* (Chen *et al.*, 2010 ▸). Figures were prepared using *PyMOL* (version 2..3.0; Schrodinger). Structure-refinement statistics are shown in Table 4 ▸.

## 3. Results and discussion   

### 3.1. Crystallographic fragment screening of the PfHsp70-x ATPase domain   

We optimized crystallization conditions for the PfHsp70-x ATPase domain, which allowed us to obtain crystals of this protein that diffracted to ∼2.2 Å resolution or better in approximately half of all drops set up. Using the fragment-screening pipeline established in the XChem laboratory at Diamond Light Source, we incubated these crystals with 233 chemical fragments from the DSI-poised (Cox *et al.*, 2016 ▸) and FragLite (Wood *et al.*, 2019 ▸) libraries and performed X-ray diffraction experiments (Supplementary Table S1). Automated processing of the X-ray data yielded 166 data sets, with 86% of these at 3 Å resolution or better (the highest resolution data set was at 1.85 Å). The overall ∼40% attrition rate of crystals that did not yield useful data could in large part be attributed to the incubation process itself, which exposes the crystals to DMSO; preliminary experiments had shown that ∼25% of crystals thus handled had worsened diffraction characteristics. The remaining losses may correspond to the fragments directly destabilizing crystal packing, for example through binding near protein contact sites in the crystals. These data suggest that performing fragment-screening assays at a lower ligand concentration or with solvents other than DMSO may reveal additional binding partners for the PfHsp70-x ATPase domain.

Diffraction data-set analysis by *PanDDA* suggested a number of putative binding events, which we followed up by full structure refinement. Three data sets revealed the unambiguous presence of ligands (six binding events in total) and these are described further in this communication (Table 4 ▸; 2*F*_o_ − *F*_c_ and *PanDDA* electron density of ligands is shown in Supplementary Fig. S1). Ligands may also have been present in a further four data sets, which have provisionally been deposited in the PDB with accession numbers 7nqr, 7nqs, 7nqu and 7nqz pending further investigation, but are not described here. We noted that although crystallization of the PfHsp70-x ATPase domain was performed in the presence of a non­hydrolysable ATP analogue, AMP-PNP, the resulting crystals trapped a hydrolysed nucleotide plus the leaving phosphate group in complex with the chaperone (Supplementary Fig. S2). Our purification process allowed ample time for the hydrolysis of any ATP molecules bound to PfHsp70-x prior to the addition of AMP-PNP, considering the basal ATPase turnover rate of this enzyme (∼0.04 s^−1^; Day *et al.*, 2019 ▸). Thus, these data suggest that our crystals trapped AMP-PNP hydrolysis events, which have also been observed previously (for example, Suzuki *et al.*, 1997 ▸), although we cannot entirely discount the possibility that some ATP molecules may have remained stably bound to the PfHsp70-x throughout the purification process. Further, the ligand PYZ reported in this study (PDB entry 7p31; ligand codes and chemical representations in Supplementary Table S1) featured weaker iodine electron density than would be anticipated, consistent with partial hydrolysis and/or radiolysis of the compound. To model this effect, we refined the fractional occupancies of the PYZ iodine and pyrazole moieties separately, resulting in iodine occupancy values that were lower by 66% and 33% (in chains *A* and *B*, respectively) compared with the pyrazole group.

**Figure 1:**
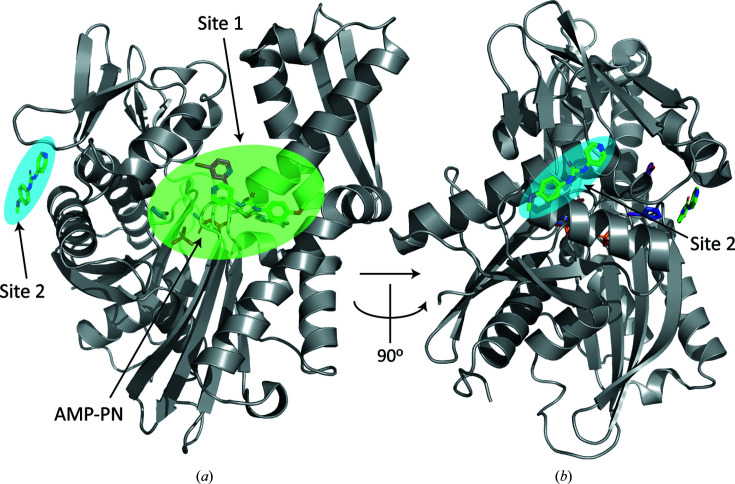
Overview of ligand binding to the PfHsp70-x ATPase domain. Shown here are the two ligand-interaction sites of the ATPase domain of PfHsp70-x identified by crystallographic fragment screening. (*a*) and (*b*) display the domain structure in perpendicular views, with the interaction sites highlighted. The main binding site (site 1) is proximal to the ATPase nucleotide-binding pocket. Ligands and the hydrolysed nucleotide bound to this domain, modelled as AMP-PN plus a leaving phosphate group, are shown as sticks.

Four out of six fragment-binding events occurred in PfHsp70-x ATPase chain *A* in the asymmetric unit of the crystals. Chain *B* featured two binding events that reproduced interactions already observed in chain *A*. The higher number of binding events noted in chain *A* is consistent with the lower average *B* factor of this chain (for example, *B* factors of 74 Å^2^ for chain *A* protein atoms in PDB entry 7ooe versus 108 Å^2^ for chain *B*), suggesting a higher level of crystal disorder and consequently lower map quality in chain *B*. Most binding events (five out of six) clustered at a site proximal to the nucleotide-binding pocket of the ATPase domain, referred to here as site 1 (Fig. 1 ▸ *a*). A further binding event (site 2) occurred on a protein ‘face’ perpendicular to site 1 (Fig. 1 ▸ *b*). We proceeded to analyse these binding sites in detail.

### 3.2. Analysis of interaction sites on the PfHsp70-x ATPase domain: site 1   

**Figure 2:**
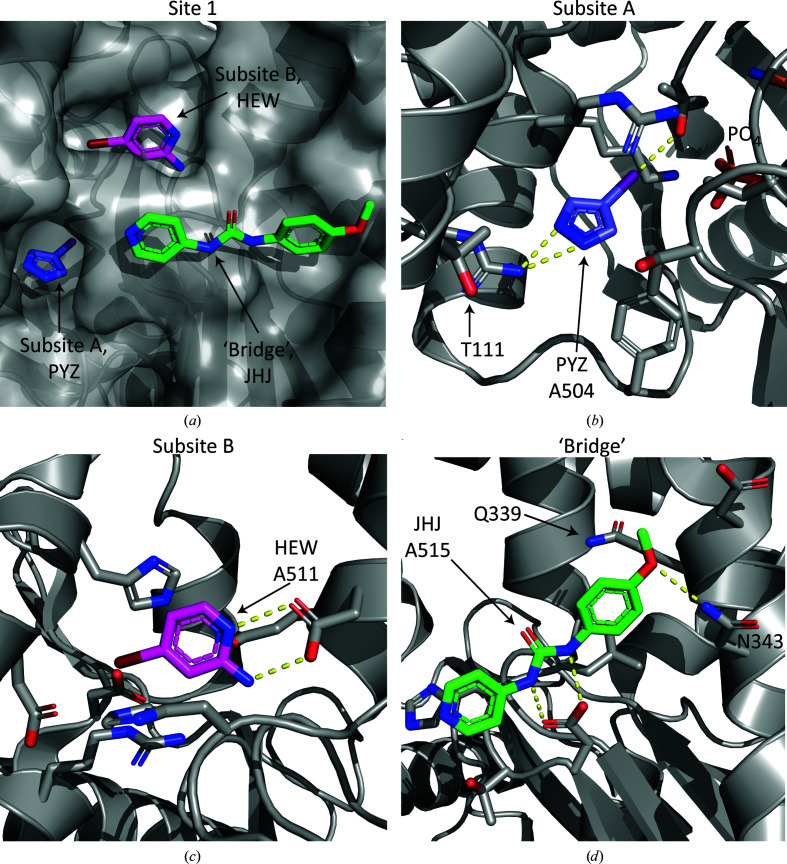
Analysis of interaction site 1. (*a*) Surface representation of the PfHsp70-x site 1, with ligands binding to this site shown as sticks. Site 1 is subdivided into three areas (subsites A and B and the ‘bridge’). (*b*–*d*) Detailed views of the three subsites. In each case the fragment ligand and protein amino acids within 4 Å are shown, with the exception of Thr111 (*b*), which is located 5 Å from the ligand. Amino-acid residues that differ between PfHsp70-x and the human erythrocytic chaperones are labelled. Yellow dashed lines indicate hydrogen bonds inferred from the structure.

Interaction site 1 of the PfHsp70-x ATPase domain can be subdivided into two subsites separated by a narrow protein ‘bridge’ (Fig. 2 ▸ *a*). Subsite A, explored by the ligand PYZ, comprises a narrow but deep pocket formed by ATPase residues Thr42, Lys100, Arg101, Arg105, Tyr179, Thr234 and Thr256 (Fig. 2 ▸ *b*). This pocket offers direct access to the departing phosphate group following ATP hydrolysis, which suggests that small molecules binding to this subsite may be able to interfere with the ATPase catalytic cycle, for example by stabilizing the hydrolysed state observed in the crystals and preventing ADP exchange. We noted that all residues forming this pocket are conserved between PfHsp70-x and the main human erythrocytic chaperones Hsp70 (HSPA1A and HSPA1B) and Hsc70 (HSPA8) (Bryk & Wiśniewski, 2017 ▸); however, residue Thr111, which is located within 5 Å of the bound fragment, is substituted by valine in the human proteins (Supplementary Fig. S3). Thus, although a challenging proposition for ligand design, exploitation of the Thr111 side-chain hydroxyl group may offer an avenue towards increasing binding specificity to the parasite chaperone.

Subsite B of site 1 comprises a large pocket, only a small part of which is explored by the ligand HEW, thereby offering significant scope for ligand growth to enhance the affinity and potentially the specificity. In our structures, direct contacts occurred between the ligand HEW and PfHsp70-x residues Asp98, Arg101, Asp115, His118, Gly233, His257, Leu258, Glu261 and Asp262 (Fig. 2 ▸ *c*). All residues within 5 Å distance of the bound ligand in subsite B are strictly conserved between the parasite and human chaperones (Supplementary Fig. S3). Subsites A and B are in close proximity (7 Å), separated by a ‘bridge’ formed by two pairs of PfHsp70-x residues engaged in hydrogen-bonding interactions (Asp115–His257 and Arg101–Thr256), which stabilize the relative position of the ATPase domain lobe IB to the rest of the protein (Day *et al.*, 2019 ▸). We judge that opening up this protein ‘bridge’ to directly engage subsites A and B using a single ligand would incur a large energetic penalty; thus, it may be more efficient to exploit both of these subsites by designing a ligand that connects them over the ‘bridge’ (Fig. 2 ▸ *a*). We observed the binding of one ligand (JHJ) in this protein area, interacting with PfHsp70-x residues Asp255–Leu258, Asp339, Gln340 and Asn343 (Fig. 2 ▸ *d*). Interestingly, two of these residues (Gln339 and Asn343) in the parasite chaperone differ substantially from those at the same position in the human counterparts (leucine and glycine or serine, respectively; Supplementary Fig. S3), raising the prospect that connecting subsites A and B via this path may also contribute to ligand specificity.

### 3.3. Analysis of interaction sites on the PfHsp70-x ATPase domain: site 2   

**Figure 3:**
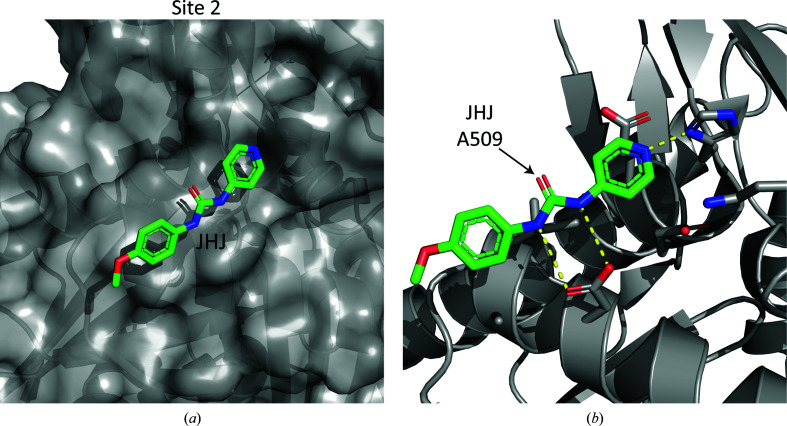
Analysis of interaction site 2. (*a*) Surface representation of the PfHsp70-x site 2, with the single ligand binding to this site shown as sticks. (*b*) Detailed view of the ligand bound to site 2 and protein amino acids within 4 Å. Yellow dashed lines indicate hydrogen bonds inferred from the structure.

Site 2 is defined by the binding of a single ligand residue (JHJ) at this position (Fig. 3 ▸ *a*). In contrast to site 1, site 2 is relatively removed from the ATP-binding cleft; thus, it is less likely that ligand interactions there would affect the catalytic activity of PfHsp70-x. Furthermore, the site comprises a shallow surface on the parasite chaperone, which suggests that engineering high-affinity ligands targeting this site may be challenging. PfHsp70-x residues in contact with the ligand JHJ include Lys130, His145, Glu147, Glu48, Asp190, Ala193 and Ile194, all of which are conserved or conservatively substituted in human erythrocytic chaperones (Supplementary Fig. S3). Thus, all evidence suggests that site 2 is less suitable than site 1 for the design of potent and specific ligands of PfHsp70-x.

## 4. Conclusions   

We showed that a relatively limited exploration of chemical space, in the form of 233 fragments, yielded a number of molecules binding to the ATPase domain of PfHsp70-x, a chaperone that contributes to the survival and virulence of the malaria parasite *P. falciparum*. Fragments bound to this domain defined two interaction sites (Fig. 1 ▸), of which site 1 is nearest to the chaperone catalytic cleft, includes a deep ligand-binding pocket and features a residue that is not conserved between human and parasite chaperones (Fig. 2 ▸). These results suggest that it may be possible to design small-molecule inhibitors of the PfHsp70-x ATPase domain that would be both potent and specific for the parasite protein.

## Supplementary Material

PDB reference: heat-shock protein 70-x ATPase domain, complexes with chemical fragments, 7ooe

PDB reference: 7oog

PDB reference: 7p31

Supplementary Table S1. DOI: 10.1107/S2053230X21007378/rf5033sup1.xlsx

Supplementary Figures. DOI: 10.1107/S2053230X21007378/rf5033sup2.pdf

## Figures and Tables

**Table 1:** Macromolecule-production information

Source organism	*Plasmodium falciparum* (isolate 3D7)
Gene	*hsp70-x* (PF3D7_0831700, UniProt accession No. K7NTP5)
DNA source	Codon-optimized synthetic DNA
Expression vector	pFLOAT
Expression host	*Escherichia coli* BL21(DE3)
Complete amino-acid sequence of the construct produced	GPAEESEVAIGIDLGTTYSCVGICRNGVVDIIANDQGNRTTPSYVAFTDTERLIGDAAKNQASRNPENTVFDAKRLIGRKFSETTVQSDMKHWPFTVKGGSDGKPMIEVSYQGEKKTFHPEEISSMVLKKMKEVAETYLGKPVKNAVITVPAYFNDSQRQATKDAGAIAGLNVLRIINEPTAAAIAYGLDKKGKGEQNILIFDLGGGTFDVSLLTLEDGIFEVKATSGDTHLGGEDFDNKLVNFCVQDFKKKNGGKDVSKNSKSLRRLRTQCEKAKRVLSSSAQATIEVDSLFDGIDYNVNITRAKFEELCMDQFRNTLIPVEKVLKDAKMDKSQVHEIVLVGGSTRIPKIQQLIKDFFNGKEPCKAINPDEAVAYGAAVQAAILSGDQSSAV

**Table 2:** Crystallization

Method	Vapour diffusion, sitting drop
Temperature (K)	293
Protein concentration (mg ml^−1^)	15
Buffer composition of protein solution	20 m*M* HEPES pH 7.4, 50 m*M* NaCl, 1 m*M* DTT, 3.5 m*M* AMP-PNP
Composition of reservoir solution	24%(*w*/*v*) PEG 1500, 20%(*v*/*v*) glycerol
Volume and ratio of drop	100 nl, 1:1 protein:reservoir solution
Volume of reservoir (µl)	40

**Table 3:** Data collection and processing Values in parentheses are for the outer shell.

PDB code	7ooe	7oog	7p31
Fragment code	JHJ, Z321318226	HEW, NCL-00023823	PYZ, NCL-00023818
Diffraction source	I04-1, DLS	I04-1, DLS	I04-1, DLS
Wavelength (Å)	0.9159	0.9159	0.9159
Temperature (K)	100	100	100
Detector	PILATUS 6M-F, Dectris	PILATUS 6M-F, Dectris	PILATUS 6M-F, Dectris
Space group	*P*2_1_2_1_2_1_	*P*2_1_2_1_2_1_	*P*2_1_2_1_2_1_
*a*, *b*, *c* (Å)	80.21, 102.68, 103.83	80.00, 101.01, 103.18	79.98, 102.57, 103.83
α, β, γ (°)	90, 90, 90	90, 90, 90	90, 90, 90
Resolution range (Å)	80.21–2.37 (2.41–2.37)	101.03–2.42 (2.46–2.42)	79.98–2.36 (2.40–2.36)
Completeness (%)	100 (99.0)	100 (99.9)	100 (100.0)
Multiplicity	6.4 (5.9)	6.4 (6.2)	6.5 (6.0)
〈*I*/σ(*I*)〉	12.9 (0.9)	8.5 (0.9)	8.0 (1.0)
Resolution at which 〈*I*/σ(*I*)〉 < 2.0 (Å)	2.6	2.7	2.6
CC_1/2_	1.0 (0.7)	1.0 (0.6)	1.0 (0.5)
*R*_r.i.m._	0.082 (1.8)	0.117 (2.1)	0.130 (2.2)

**Table 4:** Structure refinement

PDB code	7ooe	7oog	7p31
Fragment code	JHJ, Z321318226	HEW, NCL-00023818	PYZ, NCL-00023818
Resolution range (Å)	73.01–2.369 (2.39–2.37)	72.19–2.42 (2.44–2.42)	72.97–2.36 (2.38–2.36)
Completeness (%)	99.9	100	99.9
No. of reflections, working set	35507 (711)	32572 (652)	35772 (716)
No. of reflections, test set	1761 (26)	1609 (23)	1773 (28)
Final *R*_cryst_	0.204 (0.336)	0.209 (0.344)	0.220 (0.337)
Final *R*_free_	0.236 (0.410)	0.234 (0.333)	0.236 (0.364)
Cruickshank DPI	0.37	0.45	0.38
No. of non-H atoms			
Protein	5898	5885	5852
Ions ({\rm PO}_{4}^{3-}, Cl^−^, I^−^, Mg^2+^)	13	14	13
Ligand (AMP-PN)	54	54	54
Ligand (fragments)	36	16	12
Other ligands (glycerol, PEG)	109	85	43
Water	118	141	128
Total	6203	6183	6109
R.m.s. deviations			
Bonds (Å)	0.008	0.008	0.008
Angles (°)	0.98	0.98	0.97
Average *B* factors (Å^2^)			
Protein	91	85	87
Ion	131	113	104
Ligand (AMP-PN)	81	62	66
Ligand (fragments)	75	98	82
Other ligands (glycerol, PEG)	102	94	95
Water	66	65	64
Ramachandran plot			
Most favoured (%)	99	99	99
Allowed (%)	1	1	1
