# Supplementary material for: Structures of the Plasmodium falciparum heat-shock protein 70-x ATPase domain in complex with chemical fragments identify conserved and unique binding sites
Source: Acta Crystallogr F Struct Biol Commun. 2021 Jul 28;77(Pt 8):262–8. doi: 10.1107/S2053230X21007378 (PMC8329712; doi:10.1107/S2053230X21007378)
Supplement: Supplementary file 2 [file f-77-00262-sup2.pdf]

Acta Cryst F

STRUCTURAL BIOLOGY
COMMUNICATIONS

Volume 77 (2021)

Supporting information for article:

Structures of the *Plasmodium falciparum* heat-shock protein 70-x ATPase domain in complex with chemical fragments identify conserved and unique binding sites

Nada Mohamad, Ailsa O'Donoghue, Anastassia L. Kantsadi and Ioannis Vakonakis

Supporting Figures

7OOE, JHJ A509, $2F_o$-F_c, B-factor 81 Å^2, Occ 0.73, RSR 0.21

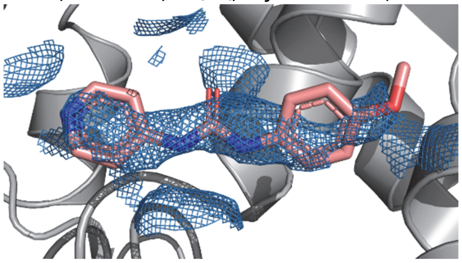

7OOE, JHJ A509, PanDDA map

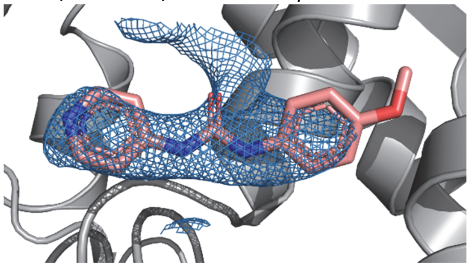

7OOE, JHJ A515, $2F_o$-F_c, B-factor 68 Å^2, Occ 0.66, RSR 0.32

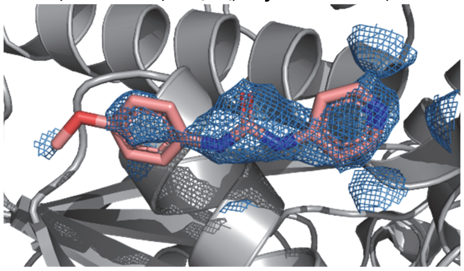

7OOE, JHJ A515, PanDDA map

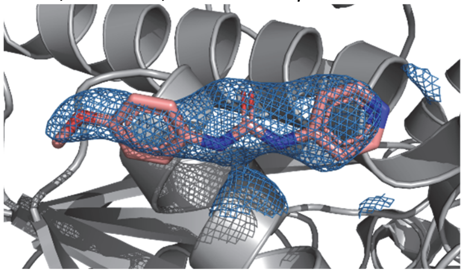

7OOG, HEW A511, $2F_o$-F_c, B-factor 70 Å^2, Occ 0.7, RSR 0.2

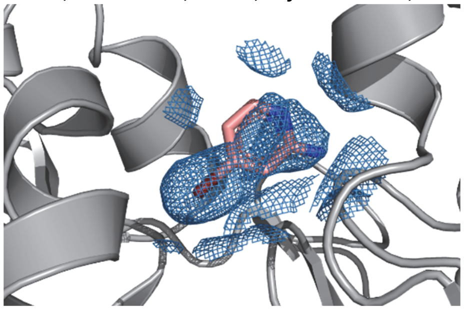

7OOG, HEW A511, PanDDA map

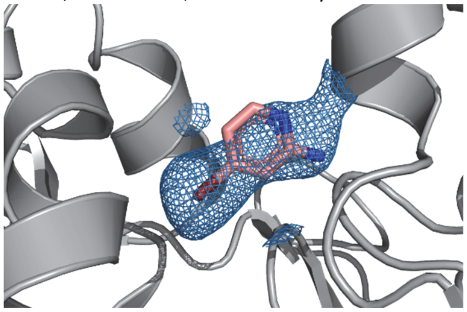

7OOG, HEW B511, $2F_o$-F_c, B-factor 125 Å^2, Occ 0.72, RSR 0.2

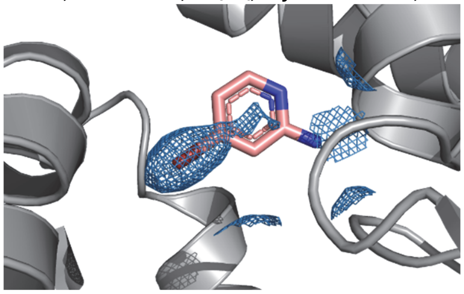

7OOG, HEW residue B511, PanDDA map

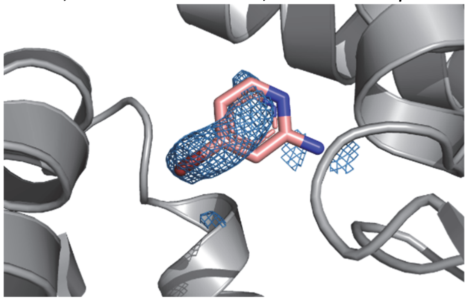

Fig. S1: Electron density of fragments. Shown here are (left column) $2F_o$-F_c electron densities and (right column) PanDDA difference densities of fragments bound to the PfHsp70-x ATPase domain, truncated at 3 Å distance from any ligand atom. The structure PDB IDs, ligand residue numbers, B-factors, occupancies (Occ) and real-space R values (RSR) are indicated. For ligand PYZ (entry 7P31, next page) reported occupancies exclude the iodine atom. $2F_o$-F_c densities were plotted at 1 r.m.s.d. level; PanDDA densities at 2 r.m.s.d. level. Figure continues on the next page.

7P31, PYZ A504, $2F_o$-F_c, B-factor 69 Å^2, Occ 0.94, RSR 0.19	*7P31, PYZ A504, PanDDA map*
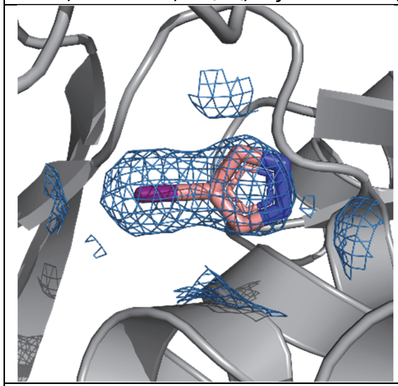	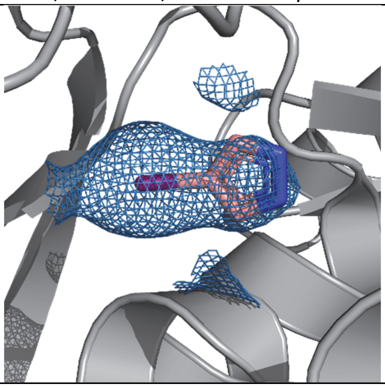
7P31, PYZ B504, $2F_o$-F_c, B-factor 96 Å^2, Occ 0.85, RSR 0.18	*7P31, PYZ B504, PanDDA map*
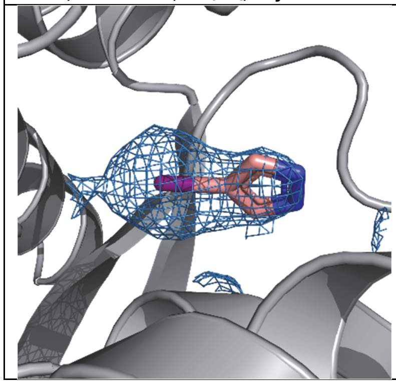	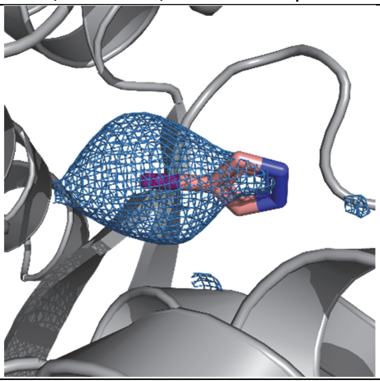

Fig. S1 continued

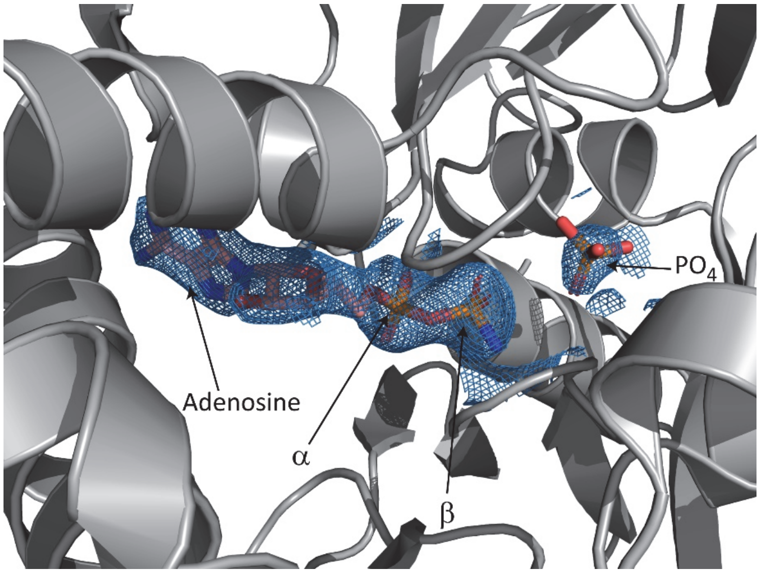

Fig. S2: Nucleotide electron density in PfHsp70-x ATPase domain crystals. Shown here is exemplar $2F_o$-F_c electron density for the bound nucleotide in PfHsp70-x ATPase domain crystals and its surrounding environment, drawn from entry 7OOE chain A. The electron density was plotted at 2 r.m.s.d. level. As seen, PfHsp70-x crystals trapped a hydrolysed nucleotide state with clear density for the leaving phosphate group.

```
Hsp70           -----------------------------MAKAAAIGIDLGTTYSCVGVFQHGKVEIIAN 31
Hsc70           -----------------------------MSKGPAVGIDLGTTYSCVGVFQHGKVEIIAN 31
PfHsp70-x       MKTKICSYIHYIVLFLIATTTVHTASNNAEESEVAIGIDLGTTYSCVGICRNGVVDIIAN 60
                               .  *:************: ::* *:****

Hsp70           DQGNRTTPSYVAFTDTERLIGDAAKNQVALNPQNTVFDAKRLIGRKFGDPVVQSDMKHWP 91
Hsc70           DQGNRTTPSYVAFTDTERLIGDAAKNQVAMNPTNTVFDAKRLIGRRFDDAVVQSDMKHWP 91
PfHsp70-x       DQGNRTTPSYVAFTDTERLIGDAAKNQASRNPENTVFDAKRLIGRKFSETTVQSDMKHWP 120
                ***************************.: ** ************:*.: .*********

Hsp70           FQVIND-GDKPKVQVSYKGETKAFYPEEISSMVLTKMKEIAEAYLGYPVTNAVITVPAYF 150
Hsc70           FMVVND-AGRPKVQVEYKGETKSFYPEEVSSMVLTKMKEIAEAYLGKTVTNAVVTVPAYF 150
PfHsp70-x       FTVKGGSDGKPMIEVSYQGEKKTFHPEEISSMVLKKMKEVAETYLGKPVKNAVITVPAYF 180
                * *  ..  .:* ::*.*:**.*:*:***:*****.****:**:***  *.***:******

Hsp70           NDSQRQATKDAGVIAGLNVLRIINEPTAAAIAYGLDRTGKGERNVLIFDLGGGTFDVSIL 210
Hsc70           NDSQRQATKDAGTIAGLNVLRIINEPTAAAIAYGLDKKVGAERNVLIFDLGGGTFDVSIL 210
PfHsp70-x       NDSQRQATKDAGAIAGLNVLRIINEPTAAAIAYGLDKKGKGEQNILIFDLGGGTFDVSLL 240
                ************.***********************:.  .*:*:*************:*

Hsp70           TIDDGIFEVKATAGDTHLGGEDFDNRLVNHFVEEFKRKH-KKDISQNKRAVRRLRTACER 269
Hsc70           TIEDGIFEVKSTAGDTHLGGEDFDNRMVNHFIAEFKRKH-KKDISENKRAVRRLRTACER 269
PfHsp70-x       TLEDGIFEVKATSGDTHLGGEDFDNKLVNFCVQDFKKKNGGKDVSKNSKSLRRLRTQCEK 300
                *::*******:*:************::**. : :**:*:  **:*:*.:::***** **:

Hsp70           AKRTLSSSTQASLEIDSLFEGIDFYTSITRARFEELCSDLFRSTLEPVEKALRDAKLDKA 329
Hsc70           AKRTLSSSTQASIEIDSLYEGIDFYTSITRARFEELNADLFRGTLDPVEKALRDAKLDKS 329
PfHsp70-x       AKRVLSSSAQATIEVDSLFDGIDYNVNITRAKFEELCMDQFRNTLIPVEKVLKDAKMDKS 360
                ***.****:**::*:***::***: ..****:****  * **.** ****.*:***:**:

Hsp70           QIHDLVLVGGSTRIPKVQKLLQDFFNGRDLNKSINPDEAVAYGAAVQAAILMGDKS     385
Hsc70           QIHDIVLVGGSTRIPKIQKLLQDFFNGKELNKSINPDEAVAYGAAVQAAILSGDKS     385
PfHsp70-x       QVHEIVLVGGSTRIPKIQQLIKDFFNGKEPCKAINPDEAVAYGAAVQAAILSGDQS     416
                *:*::***********:*:*::*****::  *:****************** **:*
```

Interaction site 1

Interaction site 2

Fig. S3: Sequence alignment of PfHsp70-x and human erythrocytic chaperones. Shown here is a Clustal Omega multiple sequence alignment of the ATPase domain of PfHsp70-x (UniProt ID K7NTP5) with the equivalent domains of human Hsp70 (P0DMV8) and Hsc70 (P11142). Residues up to 5 Å away from fragment ligands in the PfHsp70-x crystallographic structures are colour coded by binding site.
